# Retraction: An empirical assessment of financial literacy and behavioral biases on investment decision: Fresh evidence from small investor perception

**DOI:** 10.3389/fpsyg.2024.1470958

**Published:** 2024-08-01

**Authors:** Frontiers Editorial Office

The journal retracts the September 26 2022 article cited above.

Following concerns regarding the originality of the article, an investigation was conducted in accordance with Frontiers' policies. It was found that the complaint was valid and that the article should be retracted, because of an unacceptable level of similarity to an article published by Suresh G. [Suresh G. (2024). Impact of financial literacy and behavioural biases on investment decision-making. *FIIB Business Rev*. 13, 72–86. doi: 10.1177/23197145211035481].

